# Supplementary material for: Inflammatory factors and risk of lung adenocarcinoma: a Mendelian randomization study mediated by blood metabolites
Source: Front Endocrinol (Lausanne). 2024 Aug 27;15:1446863. doi: 10.3389/fendo.2024.1446863 (PMC11384989; doi:10.3389/fendo.2024.1446863)
Supplement: Supplementary file 1 [file DataSheet1.zip › supplementary files/S2/GCST90200956/forest.pdf]

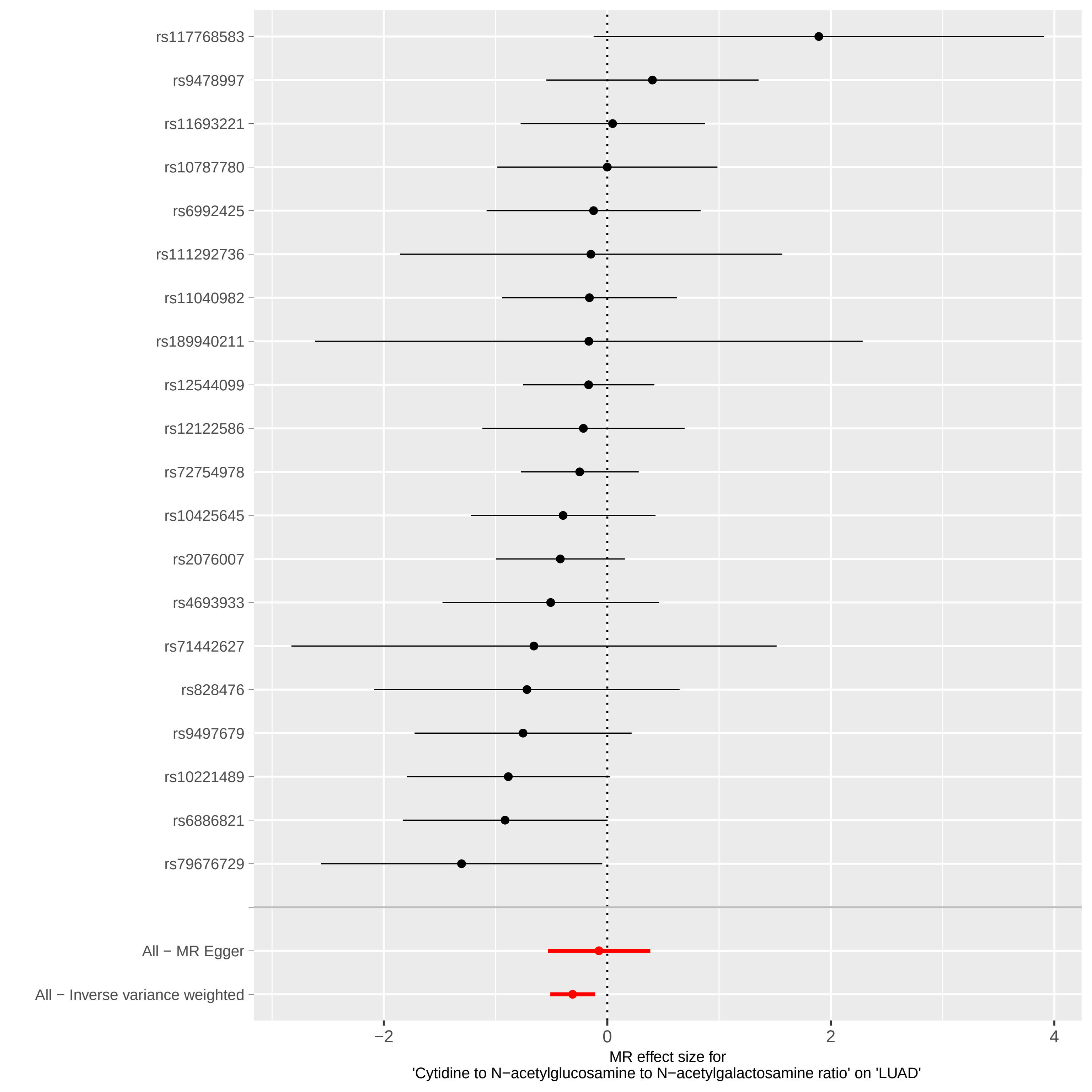
rs117768583
rs9478997
rs11693221
rs10787780
rs6992425
rs111292736
rs11040982
rs189940211
rs12544099
rs12122586
rs72754978
rs10425645
rs2076007
rs4693933
rs71442627
rs828476
rs9497679
rs10221489
rs6886821
rs79676729
All − MR Egger
All − Inverse variance weighted
−2
0
2
4
MR effect size for
'Cytidine to N−acetylglucosamine to N−acetylgalactosamine ratio' on 'LUAD'
